# Games People Play: Lessons on Performance Measure Gaming from New Zealand Comment on "Gaming New Zealand’s Emergency Department Target: How and Why Did It Vary Over Time and Between Organisations?"

**DOI:** 10.34172/ijhpm.2020.41

**Published:** 2020-03-18

**Authors:** Lisa M. Lines

**Affiliations:** ^1^Center for Advanced Methods Development, RTI International, Durham, NC, USA.; ^2^Population and Quantitative Health Sciences, University of Massachusetts Medical School, Worcester, MA, USA.

**Keywords:** Gaming, Performance Measurement, Emergency Departments, New Zealand, Healthcare Quality

## Abstract

For decades, observers have noted that gaming of performance measurement appears to be both endemic and endlessly creative. A recent study by Tenbensel and colleagues provides a detailed look at gaming of a health system performance measure—emergency department (ED) wait times—within four hospitals in New Zealand. Combined, these four hospitals handled more than 25% of the ED visits in the country each year. Tenbensel and colleagues examine whether the New Zealand ED wait time target was set appropriately and whether we can trust any performance measure statistics that are not independently verified or audited. Their thoughtprovoking examination is relevant to anyone working in quality improvement and provides a valuable set of tools for detecting gaming in performance measurement.

In *Gaming New Zealand’s Emergency Department Target: How and Why Did It Vary Over Time and Between Organisations?,* Tenbensel and colleagues provide a detailed look at gaming a health system performance measure—emergency department (ED) wait times—within four hospitals in New Zealand.^1^ Those hospitals saw more than 25% of the ED visits in New Zealand between 2006 and 2012.

## Defining the Target

When individuals arrive at an ED, they are typically triaged (assessed for how urgent their condition is and how quickly they must be seen), diagnosed, and then either treated, transferred, admitted, or discharged. Measures for describing time spent during ED visits may refer to *visit lengths* or lengths of stay ([LOS] – total time spent in the ED) or *wait time* (time until being seen by a provider). These concepts are similar, but wait time is a subset of total LOS. Once triaged and seen by the initial provider team in the ED, overall LOS may be determined by factors outside of the ED’s control, such as the availability of specialists, imaging equipment, or beds at another unit or facility. Patients waiting in the ED for resources outside the ED has been cited as the primary cause of ED overcrowding in New Zealand, although the Ministry of Health also cites problems with triage processes, insufficient ED beds, and inadequate ED staffing.^2^

## A Hard Target to Hit?

The target set by the New Zealand Ministry of Health for ED *wait times*, defined as number of minutes between when a person arrives at the ED and when that person is treated by a provider, was 6 hours or less for at least 95% of patients. This target may have been difficult for hospitals to reach. At baseline, the four hospitals studied had wildly varying performance on this measure, with anywhere from 56% to 81% of ED visits with wait times less than 6 hours. After the target was introduced in 2009, this increased to 85 to 98% of ED wait times being less than 6 hours in those same four hospitals.^1^ According to the latest government data, the average is 85% across New Zealand.^3^ As for the effects, according to one observer:

*“…the target has worked to reduce overcrowding of patients in ED by moving them on much faster to other parts of the acute hospital, or through speedier discharge from the ED. The working environment for ED staff improved as a consequence of the target…”* ^4^

Nevertheless, compared with other countries’ wait times, the achievements might seem rather poor. According to a 2010 study, at the median hospital in the United States, 87% of ED visits lasted less than 4 hours, and 93% lasted less than 6 hours.^5^ In the United Kingdom, the National Health System set a policy in 2000 to reduce ED *visit lengths*.^6^ Through concerted efforts, in 2008, 98% of ED visit lengths in the United Kingdom were 4 hours or less.^7^ Many, however, have observed that the targets in the United Kingdom were sometimes achieved without improving patient care—and in fact, may have worsened quality.^8,9^ Providers may have cut visits short or transferred patients inappropriately, known as “hitting the target, but missing the point.”^1,6,10^

## Lies, Damned Lies, and Statistics

Tenbensel and colleagues examine whether we can trust the statistics above. Gaming is endemic, yet research into variation is rare. Unfortunately, there may be as many ways to game a performance measure as there are providers.

Decades of observers have pointed out potentially problematic reactions to performance measures. Back in 1956, Ridgeway made the following observation in the journal *Administrative Science Quarterly*:

*“Quantitative performance measurements—whether single, multiple, or composite—are seen to have undesirable consequences for over-all organizational performance. The complexity of large organizations requires better knowledge of organizational behavior…”* ^11^

Hospitals are indeed complex systems in and of themselves, and national healthcare systems more complex yet. More recently, Braithwaite, writing in the *British Medical Journal*, noted the following:

*“Policy-mandated change is never given the same weight as clinically driven change. …change is always unpredictable, hard won, and takes time, it is often tortuous, and always needs to be tailored to the setting.”* ^12^

Gaming is not even the only potential hazard associated with performance measures. Writing about the UK’s national, extensive efforts to set targets and benchmarks, Mannion and Braithwaite observed 20 possible hazards, which they divided into four categories:

*“These are poor measurement (measurement fixation, tunnel vision, myopia, ossification, anachronism and quantification privileging), misplaced incentives and sanctions (complacency, silo-creation, overcompensation, undercompensation, insensitivity and increased inequality), breach of trust (misrepresentation, gaming, misinterpretation, bullying, erosion of trust and reduced staff morale), and politicisation of performance systems (political grandstanding and creating a diversion).”* ^10^

Another ED-related example cited by Mannion and Braithwaite is the introduction of “hello nurses” in some British EDs — nurses hired to greet patients within the prescribed time frame and nothing more, thereby increasing costs but not providing any actual clinical benefit.^10^ Also fitting within Mannion and Braithwaite’s taxonomy are the ways that staff and line management dealt with the intense pressure to meet the target in the four case study hospitals described by Tenbensel and colleagues. The authors describe in detail how hospitals try to appear to have reached the target, from sending patients into “black holes,” to fudging the numbers, to increasing use of short stay and observation units.^1^

Recent increases in the incidence and lengths of observation stays among patients in the United States^13,14^ have been largely explained as a result of providers trying to delay or avoid hospital admissions, whether because of lack of space on a desired inpatient unit,^15^ attempts to reduce (game) hospitalization and/or readmission rates,^16-18^ or legitimate clinical reasons.^19^ Informed by that and other research, many analysts and evaluators now analyze observation visits and outpatient ED visits separately from ED visits resulting in a hospital stay.

Beyond these kinds of ad hoc, after-the-fact adjustments, it is important to have independent verification and audits. Tenbensel and colleagues used many tools that could and should be applied elsewhere to detect implausible patterns in the data. Particularly notable is their analysis of terminal digit preference bias among the four hospitals studied. For this measure, they looked only at visits with a recorded length of stay of between 360 and 369 minutes (since the target was 6 hours, or 360 minutes). Mathematically, roughly 10% of visits in that range should have had a last digit of 0 (in other words, a recorded length of stay of 360 minutes). Tenbensel and colleagues found that terminal digit preference bias showed up after the introduction of the ED target at all four case study hospitals, with rates ranging from 11% (about what would be expected mathematically) to 38%. The higher the percentage, the more gaming. Tenbensel and colleagues’ paper plots these bias estimates in informative ways. This analysis and similar analyses should be the norm whenever analysts and policy-makers look at performance measure data.

Performance measure developers, healthcare providers and administrators, policy-makers, and researchers in the field would do well to be both humbled and encouraged by this research. Process improvement benefits have ceiling effects, and even the best measure can be improved. What does it mean for gaming to have *increased* after the benefits were realized? Would a lower target have achieved the same benefits? These and other questions are hard to answer. In the end, we are still where Ridgeway was in 1956^11^: more research is needed.

## Acknowledgements

The author would like to thank the anonymous peer reviewers for their helpful feedback and Claire Korzen, editor at RTI International, for editing assistance.

## Ethical issues

Not applicable.

## Competing interests

Author declares that she has no competing interests.

## Author’s contribution

LML is the single author of the paper.
